# Ancient and Modern Cereals as Ingredients of the Gluten-Free Diet: Are They Safe Enough for Celiac Consumers?

**DOI:** 10.3390/foods10040906

**Published:** 2021-04-20

**Authors:** Francesca Colombo, Chiara Di Lorenzo, Simone Biella, Corinne Bani, Patrizia Restani

**Affiliations:** Department of Pharmacological and Biomolecular Sciences, Università degli Studi di Milano, 20133 Milan, Italy; francesca.colombo1@unimi.it (F.C.); chiara.dilorenzo@unimi.it (C.D.L.); simone.biella@unimi.it (S.B.); corinne.bani@studenti.unimi.it (C.B.)

**Keywords:** celiac disease, gluten-free diet, *Triticum aestivum* ssp. *spelta*, *Triticum turgidum*, *Triticum monococcum*, *Avena sativa*

## Abstract

Celiac disease is an autoimmune disorder that occurs in genetically predisposed individuals after consuming prolamins from some cereals. Although the products available for celiac subjects have increased significantly in quality and quantity over the last few decades, research still focuses on identifying new ingredients to improve the nutritional, sensorial and functional qualities of gluten-free products. In terms of toxicity for people with celiac disease, there is a wide variability between ancient and modern grains. The most contradictory results are related to the role of oats in the gluten-free diet. In order to clarify the role of minor cereals (such as oat) and ancient grains in the diets of celiac patients, this review discusses recent in vitro and in vivo studies performed on those cereals for which the toxicity for celiac subjects is still controversial. According to in vivo studies, selected oat varieties could be tolerated by celiac patients. On the other hands, although some wheat-ancient grains (*Triticum monococcum*, *Triticum aestivum* ssp. *spelta* and Kamut^®^) showed a reduced in vitro toxicity, to date, these grains are still considered toxic for celiac patients. Contradictory results underline the importance of studying the safety of “unusual” cereals in more detail.

## 1. Introduction

Cereals are widely available in diets all over the world. Most cereals contain gluten, a protein complex that plays an important role in the technological properties of cereal-based products. The cereals with the highest content of gluten-like proteins are wheat, barley and rye. Wheat provides up to 50% of the caloric intake in both industrialized and developing countries, representing one of the world’s primary sources of energy [1]. Recently, pseudo-cereals (e.g., buckwheat, quinoa) have received considerable attention, since they are a good source of macronutrients, such as carbohydrates and proteins, but also of fibers, vitamins and minerals.

Gluten-related disorders (GRDs) are becoming increasingly common. The main GRDs are celiac disease (CD), non-celiac gluten sensitivity (NCGS) and wheat allergy (WA). WA is an adverse reaction that involve the immune system mediated by immunoglobulins E (IgE) [2]. NCGS is a condition characterized by intestinal and extra-intestinal symptoms associated with the consumption of gluten-containing foods in subjects who are not affected by CD or WA. The prevalence of NCGS is not clearly defined; no specific biomarkers are available for the diagnosis of NCSG. Currently, the diagnosis is based on exclusion criteria (absence of CD and WA) and positive diagnostic challenges (double-blind, placebo-controlled gluten challenge) [3].

The role of gluten is well documented in celiac disease (CD), including dermatitis herpetiformis. CD, one of the most common lifelong diseases worldwide, showed a global prevalence of 1.4%, based on serologic tests, and 0.7%, based on in vivo tests (biopsy) [4]. 

CD is an autoimmune disorder, occurring in genetically predisposed subjects, having the necessary co-factor of the consumption of toxic prolamins [5,6]. Of these, the most toxic proteins are wheat gliadins (a portion of gluten); however, rye secalin and barley hordein show similar toxicities in celiac subjects [6]. After deamidation, due to the action of transglutaminase, peptides stemming from the proteolysis of gliadin and other toxic prolamins, bind human leukocyte antigen (HLA)-DQ2 or DQ8, thus leading to inflammation [5]. These complexes are associated with the production of auto-antibodies generally belonging to the immunoglobulins A (IgA) class [7]. 

Celiac disease is characterized by a condition of malabsorption correlated with enteropathy characterized by small-intestinal villous atrophy. In addition to the intestinal symptoms, CD could be characterized by different extra-intestinal complications, such as bone and skin diseases, microcytic anemia (due to iron deficiency), endocrine disorders, and neurologic deficits [5].

At present, the total exclusion of toxic prolamins from the diet is the only accepted treatment for patients with CD. Gluten is an important agent in baking products, contributing to dough viscosity and elasticity [8,9]. As a consequence, different studies underline the limitations of gluten-free products (in particular, gluten-free bread), in terms of technological and sensorial properties [8]. In this contest, the research still focuses on identifying new ingredients to improve the nutritional, sensorial and functional qualities of gluten-free products. The safety of cereals used in gluten-free products, thus, need to be monitored continuously, since in some cases, it depends on the variety considered (as discussed in paragraph 2.4. for oat) [10,11,12,13]. 

**Figure 1:**
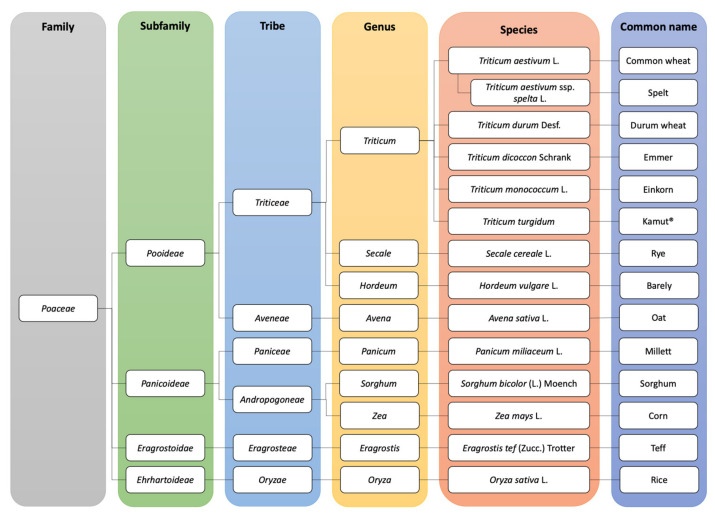
Botanical classification of cereals [14,15].

Figure 1 shows the botanical classification of cereals. All cereals belong to the *Poaceae* family. The main toxic cereals (e.g., wheat, rye, and barley) belong to the subfamily of *Pooideae* and contain CD-eliciting epitopes. On the other hand, rice, millet and corn are safe for CD patients.

Table 1 summarizes the main cereals and pseudocereals currently allowed or not allowed in the gluten free diet (GFD) [16].

Rice and corn are the most common alternatives to gluten-containing grains in the GFD. Among the minor cereals, particular attention has been given to oat, which has been widely used in Northern Europe. *Avena sativa* L. has interesting nutritional properties [17]; it presents a high fiber content and a low glycemic index compared to other cereals or pseudocereals (e.g., buckwheat, quinoa, sorghum, and teff) [18] and could improve the palatability of gluten-free products. Oat was once excluded from the GFD in Mediterranean countries but recently the EU included oat as a gluten-free ingredient [19], although its inclusion in the CD diet is still a matter of debate.

Table 1 includes Kamut^®^, a patent referring to *Triticum (T.) turgidum*, spelt (*T. aestivum* ssp. *spelta*) and einkorn wheat (*T. monococcum*) as toxic cereals.

Interest in the consumption of ancient grains has recently increased and various in vitro and ex vivo methods have been used to evaluate the safety of these cereals for celiac patients. 

Table 2 shows the main tests used to assess the toxicity of cereals in CD. K562(S) and Caco-2 cells are the principal cell lines used, and various outcomes (such as the agglutination ratio, transepithelial electrical resistance and expression of tissue transglutaminase) are considered.

These tests often provide conflicting information on the safety of these grains for celiac patients. For example, studies on spelt underline the large variations among accessions, in terms of toxicity for celiac subjects [20,21,22]. Although still excluded from the GDF diet, recent studies have suggested that the wheat *T. monococcum* presents a lower number of immunogenic peptides and a higher in vitro digestibility compared to the hexaploid common wheat [23]. All these findings have led to an increasing interest in the study of ancient grains in terms of safety for celiac patients.

The present paper reviews the recent in vitro and in vivo studies performed on those cereals whose toxicity for CD is still controversial.

## 2. Methods

A literature revision was performed to summarize the data at our disposal and clarify the safety of some discussed cereals (ancient grains and minor cereals, such as oat) in the gluten-free diet. PubMed, MEDLINE, Embase, and CAB-Abstract were searched (from database inception to November 2020) using the terms “Spelt or *Triticum aestivum* ssp. *spelta*”, “Kamut^®^ or *Triticum turgidum*”, “Einkorn or *Triticum monococcum*”, “Oat or *Avena sativa*” in combination with “celiac disease” and “gluten”.

The search by title and abstract produced 335 papers. The inclusion criteria were based on the selection of papers reporting in vitro and in vivo studies assessing the safety of the cereals considered for celiac patients. Reviews, duplicates or papers not focused on the required topic were excluded. A final total of 52 papers were selected (Table 3).

## 3. Results and Discussion

### 3.1. Triticum monococcum

Some studies suggest a possible difference in the number of T-cell stimulatory peptides in wheat varieties [24]. Due to its simpler genome and to the presence of prolamins that are more susceptible to gastrointestinal digestion [23], some authors have suggested that *T. monococcum* may contain a lower number of epitopes and toxic peptides. In vitro and ex vivo studies have provided contradictory results on the safety of *T. monococcum* for patients suffering from CD (Table 4), suggesting that its use in GFD should be excluded or at least considered with extreme caution.

Most studies on the safety of cereals in celiac patients investigate the products after peptic–tryptic digestion (PTd) [25]. The outcomes most frequently considered are: agglutination, the secretion of cytokines or the alteration of transepithelial electrical resistance (TEER) in cell models, such as Caco-2 cells [26,27,28], K562 (S) cells [26,29], or gliadin reactive T-cell lines [23,30,31,32,33]. 

Although PTd products from *T. monococcum* may determine a T cell response [30,31], a more extensive proteolysis by the brush border membrane enzyme (BBM) seems to be responsible for a reduction in its immune stimulatory properties [23,32]. The ex vivo experiments, performed using duodenum biopsies, showed similar results: no morphological change was observed by Pizzuti et al. (2006) in biopsies cultured with *T. monococcum* [34]. On the other hand, a reduction in immunotoxic potential was observed when tests were performed in ex vivo organ culture after extensive digestion by BBM enzymes [32]. Gianfrani et al. (2012) tested two different varieties of *T. monococcum* on jejunal biopsies, and both samples caused intraepithelial T cell infiltration and lamina propria T cell activation [30].

The ability of *T. monococcum* to trigger toxic effects in CD was also studied in vivo (Table 5). In an intervention study aimed to evaluate the safety of the daily administration of *T. monococcum* for 60 days, the concentration of CD-related antibodies changed from negative to positive in 60% of patients [35]. Zanini et al. investigated the gastrointestinal events associated with *T. monococcum* consumption and concluded that one single low dose of this cereal is generally well tolerated by celiac patients [36]. Another study underlined that *T. monococcum* elicited a reduction in T-cell response compared to *T. aestivum* [36,37].

Generally speaking, 75% of the in vitro and ex vivo studies listed in Table 4 suggest that *T. monococcum* is less toxic for celiac patients than traditional varieties. On the other hand, the intervention studies, considering both short and chronic administration of *T. monococcum,* produced negative or inconclusive results. In conclusion, although *T. monococcum* at present is not suitable for GFD, further studies are in progress which will produce data for further evaluation [35].

### 3.2. Triticum aestivum ssp. spelta

In the scientific literature *Triticum aestivum* ssp. *spelta* was indicated as a cereal potentially tolerated by celiac subjects, thanks to its genotype being poor in prolamins [26]. At present, only the proteomic approach has been used for the assessment of this cereals; in vitro studies are needed before in vivo trials can be planned. On these bases, this review will list and discuss the papers that support tolerance using a proteomic approach.

*Triticum aestivum* ssp. *spelta* is usually considered toxic for celiac patients, although its high genetic variability in the germplasm could lead to the development of varieties with a lower toxicity for CD patients. Several studies, based on proteomic approaches, have investigated α-gliadin expression in spelt varieties (Table 6). They have underlined a high expression variability among varieties and epitopes [21,22,38]. In addition, no influence on these parameters by environmental factors, such as the harvest year and N fertilization was observed [20,22]. In a study by Asledottir et al. (2020) after an ex vivo digestion, the ancestral cereals (*T. monococcum*, *T. aestivum* ssp. *spelta* and *T. diccocum*) released fewer T-cell epitope-containing peptides than the common wheat varieties [25]. However, at present, ancestral wheat is considered toxic for celiac patients.

Few in vitro studies have been conducted to evaluate the presence of cytotoxic prolamins for celiac disease in spelt. The potential immunogenicity of spelt and wheat accessions, evaluated using A1 and G12 monoclonal antibodies (A1 antibodies recognize the sequence QPQLPY and G12 recognizes the sequence QLPYPQP, which are present in DQ2.5-glia-α1a, DQ2.5-glia-α1b and DQ2.5-glia-α2 immunogenic epitopes), highlighted the great variability in terms of reactivity in both subspecies: accessions with a lower reactivity were found in both subspecies [20]. Regarding cellular tests, spelt wheat showed toxic effects on Caco-2 and K562 (S) cells. Increasing amounts of nitric oxide and transglutaminase-2 (TG-2) were observed in Caco-2 cells after treatment with spelt prolamins [26].

In summary, although a high expression variability among epitopes and *Triticum aestivum* ssp. *spelta* accessions has been found, the in vitro studies showed toxic effects and, for the moment, spelt wheat cannot be considered safe for celiac patients.

### 3.3. Kamut^®^

The taxonomy of Kamut^®^ is controversial, and it has been classified as *T. turgidum polonicum, T. turgidum turanicum*, or *T. turgidum durum*. However, recently, Khlestkina et al. defined Kamut^®^ as a hybrid between *T. durum* and *T. polonicum* [39]. Today, Kamut^®^ is generally considered an ancient cereal correlated to the durum subspecies [40]. In the last few years, ancient wheats have been re-introduced into agriculture practices in order to maintain biodiversity. It has also been suggested that some ancient varieties may be less toxic for people suffering from food intolerances or allergies [24,33]. In order to evaluate this controversial hypothesis, several authors have tested the ancient wheat Kamut^®^ in terms of its CD-immunogenic properties.

Various peptides, derived from Kamut^®^ α-gliadins (such as the peptides p56–75), stimulate the T cell response [42,43]. In addition, other peptides (e.g., p31–49) activate the innate immune system [44]. In this context, several ELISA and western blot analyses have been carried out using specific monoclonal antibodies (mAbs—anti-p31–49 and mAbs anti-p56–75). Kamut^®^ samples always showed an antibody–antigen positive reaction resulting in a similar toxicity to that obtained with modern wheats [40,44].

Small intestinal gluten-specific T-cell lines from celiac patients were tested with ancient and modern varieties in proliferation assays to evaluate whether the different varieties of wheat (including Kamut^®^ ) were equally toxic to celiac patients. All the wheat varieties tested caused heterogeneous small intestinal T-cell responses, independent of their ancient/modern origin or ploidy [31].

In addition, the analysis of ancient grains, such as einkorn, emmer, Kamut^®^, rye, teff and sorghum, using an untargeted mass spectrometric method and a reverse phase-HPLC highlighted the presence of celiac epitopes in wheat-related ancient grains (e.g., einkorn, emmer, and Kamut^®^). Although differences in gliadin protein composition were found between ancient grain species [45], on the basis of the current data, Kamut^®^ is toxic for celiac patients and should be excluded from the diet of celiac subjects.

### 3.4. Avena sativa L.

Among minor cereals, particular attention has been paid to oat. *Avena sativa* L. is a good source of vitamins (B complex), protein, fat, minerals and soluble fiber β-glucan. Oat (if considered safe) could, thus, increase the nutritional value of GFD and improve the palatability of gluten free products [17]. The Commission Regulation EU 828/2014 [19] allows the inclusion of oat in the gluten-free diet only if this cereal have been specially produced, prepared and/or processed in order to avoid contamination from other gluten-containing cereals (gluten content < 20 ppm). In addition, the regulation states that “Most but not all people with intolerance to gluten can include oats in their diet without adverse effect on their health”. For this reason, the inclusion of oat in GFD is still a matter of debate in the scientific community.

The different toxicities of cereals (wheat, barley and rye), non-toxic cereals (rice and corn) and oat (debated) for celiac subjects could be explained by the significant taxonomic differences between these cereals. Oat and toxic cereals belong to the same family of *Poaceae* but to different tribes (toxic cereals to *Triticeae* and oat to *Aveneae*) which may explain the different contents of toxic prolamins in these cereals. Oat prolamins (avenins) represent 10–15% of the total protein content, whereas prolamins of toxic cereals (wheat, barley and rye) constitute 30–50% of total protein.

In addition, the prolamins of toxic and non-toxic cereals show differences in amino acid composition: the percentages of proline and glutamine residues (both involved in the pathogenesis of CD) in toxic prolamins (from rye, barley and wheat) are 20% and 36%, respectively. Avenins contain a similar percentage of glutamine (34%) but a lower content of proline (10%) [46].

Contradictory results have been obtained regarding the role of oat in the GFD. Some studies have underlined the presence of different contents of oat immunoreactive epitopes in different varieties, while others explain the differences as a consequence of the contamination of oat products by other gluten-containing cereals [47,48]. Oat contamination has been tested using different in vitro techniques such as ELISA and immunoblotting. Several authors have reported that oat could be contaminated by gluten, and in a study performed on 133 commercial oat samples in Canada, 88% of the samples had a gluten concentration above 20 ppm [49]. Similar results were obtained in other studies for oat samples collected in Europe, the United States and Canada [50,51]. The origin of contamination may differ, depending on the field to the packaging [49]. All these studies underline that any oat varieties used for the celiac population must be free from gluten contamination.

Cross-contamination is not the only source of toxicity for celiac patients; in fact, oat avenins show a great variability which influences the immunoreactivity of peptides at the intestinal level [10].

To define the safety of oat for celiac subjects, several in vitro experiments have been performed. Some studies found no in vitro activities related to CD pathogenesis. Picarelli et al. (2001) examined the anti-endomysial antibody (EMA) production in supernatant fluid in cultured duodenal mucosa specimens from CD patients after oat and gliadin treatment [52]. EMAs were always detected after challenge with gliadin, but not after culture with digested avenins. The activities of two oat varieties (*Avena genziana* and *Avena potenza*) were tested in vitro in terms of the phosphorylation of extracellular signal-regulated kinase and TEER, in Caco-2 cells treated with PTd products from the two oat varieties and from gliadin. No negative in vitro activities related to CD were observed with these two specific varieties [53].

Other studies observed an in vitro toxicity of avenins [11,12,13,54,55,56]. Silano et al. (2014) evaluated the ability of different oat cultivars to activate the gliadin-induced transglutaminase-2 (TG2)-dependent events using in vitro models of CD [11]. They observed that some oat cultivars elicited these events, whereas other varieties did not [11].

Some avenin-reactive T-cell lines (obtained from nine celiac patients) recognized avenin peptides in the context of HLA-DQ2 [55]. Some oat peptides also stimulated the circulating dendritic cells, which are an important connection between innate and immune responses in CD pathogenesis [54].

The different levels of toxicity observed with oat varieties [12,13] highlight the importance of screening oat varieties by in vitro tests, in order to assess their safety before starting clinical trials [11].

The sera reactivity of CD patients versus oat has also been investigated. The antibody responses of children with CD against oat prolamins were tested in 34 subjects and compared with 47 control sera. Children with CD had significantly higher levels of IgG and IgA versus avenins, compared to the control group [57]. Vainio and Varjonen (1995) also detected the reactions of CD patients’ serum to oat; however, some individual specificity was observed [58]. On the other hand, in a study by Guttormsen et al. (2008), using the serum of 136 CD adult patients (60% of subjects had consumed oat as part of their GFD), no significant differences were found in IgA against oat in oat-eating CD patients and the control group (non-oat-eating CD subjects) [59].

Some intervention studies (Table 7), performed on children (aged between 0.7–17.2 years) indicated that oat could affect the health of CD patients, leading to gut mucosal inflammation, with a possible risk for future complications [60]. 

As indicated by the immune status of the intestinal mucosa, some pediatric CD patients seem to be reactive to oat [61]. Concerns regarding the safety of oat also remain for adult CD patients. After a 12-week intervention study conducted with 50 g oat/day, one patient developed villous atrophy and dermatitis after oat consumption, and 26% of patients showed positive levels of interferon γ mRNA after the oral challenge [62].

Other clinical trials have shown that patients with CD (both adult [63,64,65,66,67,68,69] and children [62,70,71,72,73,74,75]) can safely consume medium/high amounts oat when uncontaminated by gluten (Table 8).

A double-blind, placebo-controlled, crossover study evaluated the long-term (15-month trial) consumption of oat in children (177 patients, 4–14 years of age) with celiac disease. Children were randomly assigned to Group 1 (six months of a GFD plus A products, three months of washout with a standard GFD, and six months of GFD plus B products) or Group 2 (six months of a GFD plus B products, three months of washout, and six months of GFD plus A products). A and B products consisted of gluten-free foods (such as pasta, flour and biscuits) containing either purified oat or placebo. For this study two oat varieties (“Irina” and “Potenza” *Avena sativa)* were selected, after a preliminary screening in vitro for immunoreactivity [13]. Clinical, serological and intestinal permeability data were collected at baseline and after six, nine and fifteen months.

No statistically significant effect was observed for clinical, serological, and intestinal permeability biomarkers after the treatment. This study highlights that the long-term consumption of pure non-reactive oat products is safe for children with CD [75].

In a large cross-sectional study, different outcomes were compared between celiac patients (n = 169) on a GFD with or without oat. A total of 82% of the participants interviewed consumed oat. Oat consumers and non-consumers did not differ in dietary adherence, prevalence of symptoms, positivity for antibodies, histological recovery after one year, osteoporosis/osteopenia, or fractures. The oat consumers showed better general health scores [76]. In addition, oat could improve the nutritional and sensory quality of a gluten free diet. In fact, patients that consumed oat had a significantly higher daily intake of fiber [66] and thiamine and Zn [68] than those who did not consume oat. Regarding the sensory quality of the diet, the majority of CD patients like oat in their diet [63,71].

Based on current evidence, celiac patients can safely consume oat, but only after appropriate screening in order to exclude the immunoreactivity of the selected varieties.

## 4. Conclusions

Although the EU 828/2014 reported that celiac subjects should avoid wheat (e.g., *Triticum* species, spelt and Kamut^®^), rye and barley [19], recent studies underline the presence of variability, in term of immunostimulating epitopes, between ancient and modern grains. Minor cereals (such as oat) and ancient cereals have received considerable attention as alternatives for the formulation of gluten-free products. However, their toxicity for celiac patients is still debated.

Some ancient wheat varieties would appear to be less toxic for celiac patients. For example, *T. monococcum* seems to contain a lower number of toxic peptides and its prolamins are more susceptible to gastrointestinal digestion. Although some wheat-related ancient grains, such as *T. monococcum*, *Triticum aestivum* ssp. *spelta* and Kamut^®^, show a reduction in in vitro toxicity, the studies presented in the literature highlight the presence of immunostimulating epitopes. Therefore, these grains are currently considered toxic for celiac patients. Some of these varieties, with a reduced ability to activate the immune response in CD mucosa, could be useful in reducing the incidence of CD, but future studies are needed to confirm this.

In 2009, oat was permitted in the EU as a gluten-free ingredient; however, the contamination of oat products by other gluten-containing cereals is only one of the problems associated with the safety of this cereal for celiac patients. In fact, some in vitro studies suggest that oat avenins show great variability that influences their immunoreactivity at the intestinal level. These results underline the importance of screening the safety of oat varieties by in vitro tests before starting clinical trials.

Although some trials describe the reduced tolerability of oat in a fraction of CD patients, the majority of in vivo studies highlight that selected uncontaminated and nonreactive varieties can be safely included in the GFD.

This paper has reviewed the main in vitro and in vivo studies performed on those cereals for which the toxicity for CD is still controversial. It is evident that this review shows limitations; among others, is the fact that the various experimental protocols were not compared. On the other hand, the aim was a collection of available data from the scientific literature without intervening on the significance or otherwise of the various experimental approaches.

Contradictory results remain which highlight the importance of studying the safety of “unusual” cereals in more detail in order to prevent adverse effects in celiac patients. Oat is an exception, as selected varieties of this cereal have proven to be well tolerated in a long-term clinical study.

## Figures and Tables

**Table 1:** Cereals and pseudocereals and their inclusion in the GFD.

Allowed	Not Allowed
Cereals	
Corn	Wheat (Spelt, semolina, durum)
Rice	Rye
Sorghum	Barley
Oat *	Kamut^®^
Pseudocereals	
Buckwheat	
Quinoa	
Amaranth	

* Still a matter of debate.

**Table 2:** In vitro and ex vivo tests most frequently used to evaluate the toxicity of cereals in CD.

Test	Outcome
K562(S) cells	Agglutination test: K562(S) cells agglutinated after contact with gluten. The agglutination ratio is strongly correlated with cereal toxicity
Caco-2 cells	Inhibition of cell growth, correlated with toxic cereals
	Activation of cell apoptosis
	Nitric oxide (NO) release: Cao-2 cells produce NO after exposure to toxic peptides
	Alteration of transepithelial electrical resistance (TEER): Toxic cereals influence the barrier integrity with a consequent decrease in TEER
	Expression of tissue transglutaminase (TG II): increasing amounts of TG II are associated with toxic cereal exposure
	Cytoskeleton reorganization: gliadin-derived peptides induce early actin reorganization
	Zonulin release: Gliadin peptides induce zonulin release correlated with the alteration of epithelial permeability
Biopsy specimens from the duodenum are processed for ex vivo organ culture	Conventional histology (e.g., enterocyte height)
	Immunochemical studies (e.g., expression of HLA-DR, number of CD3+ T cells; Interferon-γ secretion)
T cell line (generated from duodenal biopsies)	Cell proliferation test: Evaluates the immune stimulatory properties of cereal samples
	Immunochemical studies (Interferon-γ secretion)

**Table 3:** Studies collected from database searches. Those selected are in brackets.

Key Words	Celiac Disease	Gluten	Total
Spelt or *Triticum aestivum* ssp. *spelta*	17 (6)	27 (0)	6
Kamut® or *Triticum turgidum*	7 (4)	4 (0)	4
Einkorn or *Triticum monococcum*	20 (12)	28 (2)	12
Oat or *Avena sativa*	70 (11)	162 (19)	30

**Table 4:** In vitro and ex vivo studies to evaluate the toxicity of *Triticum monococcum* for celiac subjects.

Objective of the Study	Methods	Main Outcomes	Ref.
***Lower toxicity***			
To evaluate the toxicity of gliadin peptides from 10 varieties of *T. monococcum.*	The peptic–tryptic digestion products (PTd) of alcohol-soluble proteins from samples were tested for their agglutinating activities in K562(S) cells.	No agglutination was observed in K562(S) cells.	[29]
To evaluate the T-cell stimulatory capacity of different wheat species (including *T. monococcum*).	Gluten was extracted from different ancient wheat species and screened for T-cell stimulatory gluten peptides.	The intestinal T-cell response was different for the diploid species considered. The sequence of gliadin 33 mer (the most toxic) was not observed in diploid einkorn.	[33]
To determine the toxicity of *T. monococcum* on small intestinal mucosa through an organ culture system.	29 distal duodenum biopsies (12 from treated celiac patients, 17 from control subjects) were cultured for 24 h with 1 mg/mL of gliadin from *T. aestivum* (bread) or from *T. monococcum*. Tests included conventional histological examination, immunohistochemical detection of CD3 + IELs, HLA-DR and the IFN-γ.	*T. monococcum* gliadin did not determine any significant morphological changes in celiac subjects, HLA-DR overexpression in the crypt epithelium, increased number of CD3 + IELs nor significant IFN-γ response.	[34]
To evaluate the cytotoxicity of prolamins for celiac disease in different species from ancient wheat (*T. aestivum* ssp. *spelta*, *T. monococcum* and *T. turgidum* ssp. *dicoccum*).	Cytotoxicity was evaluated in term of activation of apoptosis, inhibition of cell growth, release of nitric oxide, alteration of TEER and detection of TG II on Caco-2/Tc7 and K562(S) cell agglutination.	The PTd from *T. monoccum* wheat did not show any negative effects on Caco-2/TC7 and K562(S) cells.	[26]
To investigate the toxicity of *T. monococcum* and *T. aestivum* cultivars after treatment with the products from PTd or extensive digestion with brush border membrane enzymes.	T-cell lines or jejunal biopsies from celiac patients were tested to evaluate the immunostimulatory properties of digested samples.	The T-cell response profiles due to PTd products from *T. monococcum* and *T. aestivum* were comparable. However, extensive gastrointestinal treatment drastically reduced the immune stimulatory properties of *T. monococcum* gliadin. MS-based analysis showed that several *T. monococcum* peptides were hydrolyzed during gastrointestinal digestion.	[32]
To investigate the biological effects of *T. monococcum* on human Caco-2 intestinal epithelial cells.	The effects of gliadin-derived peptides from *T. monococcum* (*ID331)* were tested on epithelial permeability, zonulin release, viability, and cytoskeleton reorganization. *Triticum aestivum* was used as a positive control.	ID331 gliadin did not alter the parameters evaluated in the Caco-2 cell monolayers. Some ID331 peptides showed a protective action, reducing the damage due to *Triticum aestivum* gliadin on cytoskeleton reorganization and cell viability.	[27]
To compare the immunological properties of gliadins from two *T. monococcum* cultivars (Hammurabi and Norberto-ID331) versus *Triticum durum* (Adamello).	The product from in vitro digestion with brush border membrane enzymes was tested for its effect on IFN-γ production in T-cell lines from celiac disease subjects.	The ability of gliadins from *T. monococcum* of both cultivars to activate T cells was reduced by gastrointestinal digestion, determining a lower toxicity in celiac patients (*p* < 0.05).	[23]
To evaluate the toxicity of two ancient *T. monococcum* varieties (ID331 and Monlis) for consumers with gluten related disorders.	The effect of peptides from digestion on Caco-2 cells was evaluated. *T. aestivum* was used as a positive control.	Differences between the varieties included were observed: (1) ID331 did not enhanced cell permeability and did not induced zonulin release in Caco-2 monolayers, (2) Monlis showed detectable toxicity.	[28]
To identify peptides stimulating T cells after ex vivo digestion of ancestral (including *T. monococcum*) and common wheat using human gastrointestinal juices.	Wheat porridge from ancestral and common cereals was digested using a static ex vivo model (240 min) and analyzed with high-performance liquid chromatography/ electrospray ionization tandem mass spectrometry (HPLC-ESI MS/MS).	Ancestral wheat, compared to common wheat, released fewer immunogenic peptides after ex vivo digestion. However, ancestral wheat was still highly toxic for celiac patients.	[25]
***Toxic response***			
To compare the immunological properties of *T. monococcum* versus *T. aestivum*.	PTd products from samples were tested for their effects on IFN-γ production and proliferation of intestinal gliadin-specific T cell lines and clones. The effects of PTd products from gliadin on innate and adaptive immune response were evaluated in organ cultures of jejunal biopsies from 28 celiac patients by immunohistochemistry.	*T. monococcum* samples induced IFN-γ production and proliferation in celiac mucosal T cells. A different activation of innate immune pathways was observed between the two lines of *T. monococcum* tested but both were toxic for celiac patients.	[30]
To study the toxicity of *Triticum* accessions with different origin (ancient/modern) and ploidy (di-, tetra-hexaploid).	T-cell lines, generated from 13 celiac patients, were tested with wheat samples in proliferation assays.	All varieties of wheat, regardless of ploidy or ancient/modern origin, determined heterogeneous responses considering a wide range of stimulation indices.	[31]

PTd: Peptic–tryptic digestion product; IELs: intraepithelial lymphocytes; IFN-γ: interferon-gamma; TEER: transepithelial electrical resistance; TG II: tissue transglutaminase.

**Table 5:** In vivo studies to evaluate the toxicity of *T. monococcum* for celiac subjects.

**Study Details**	**Objective of the Study**	**Protocol**	**Main Outcomes**	Ref.
Single blind, cross-over study; 12 celiac patients (mean age: 40.9 years) on GFD for at least 12 months.	To investigate the safety of a single dose of gluten from Tm.	Follow up: day 0, 14 and 28. Dose: 2.5 g of Tm, rice or pure gluten (Amygluten). End-points: Changes in intestinal permeability (measured with the urinary lactulose/rhamnose ratio (L/R ratio) and the occurrence of adverse gastrointestinal events (World Health Organization (WHO) scale).	The oral challenge with the three cereals did not determined changes in urinary L/R ratio. In all cases, occurrence of gastrointestinal events was graded as: (1) “mild” or “moderate” with Tm and rice, (2) “severe” or “disabling” with Amygluten (*n* = 4).	[36]
Intervention study; 5 patients (F/M: 4/1, age: 19–44 years).	To evaluate the safety of chronic daily intake of Tm.	Protocol: Administration of 100 g/day Tm biscuits for 60 days. End-points: Symptoms (recorded with the Gastrointestinal Symptom Rating Scale questionnaire—GSRS), CD-related serology (T0, T30 and T60 days) and duodenal biopsy (T0 and T60).	No difference in GSRS score were observed at T0 and T60. All patients had Marsh II lesion at T0; 4 had Marsh III and 1 had recurrence of dermatitis herpetiformis at T60. The antibodies CD related to converted from negative to positive at T60 in 3 patients.	[35]
Oral challenge; 17 subjects with CD (median age: 13 years).	To evaluate the gluten-reactive T-cells elicited by diploid and hexaploid wheat in CD subjects after short oral challenge.	Protocol: For 3 days, patients consumed sandwiches made with Tm, or Ta flour, corresponding to 12 gr of gluten/day. End-points: Quantification of IFN-γ-secreting T-cells subjects using EliSpot and the expression of inflammatory cytokines/receptors (IL-12A, IL-15, IL-18RAP, IFN-γ) by qPCR.	Tm (*p* > 0.05) compared to Ta did not induce significant cell mobilization (*p* > 0.05). The group consuming Ta showed an increased mRNA expression for IL-12A and IFN-γ compared to the group consuming Tm ( *p* < 0.05).	[37]

CD: Celiac disease; GFD: Gluten free diet; Ta: *Triticum aestivum*; Tm: *Triticum monococcum.*

**Table 6:** Toxicity of spelt (*T. aestivum* ssp. *spelta*) based on proteomic approach.

Aim of the Study	Methods	Main Outcomes	Ref.
To evaluate the toxicity of spelt wheat for celiac subjects.	Spelt wheat and *T. aestivum* L. were compared by analyzing α-gliadin N-terminal portions.	The identity of spelt and bread wheat was confirmed by the N-terminal sequences of alpha-gliadins (from position 3 to 56).	[41]
To investigate and compare the genetic diversity of gliadin transcripts from spelt and bread wheat.	Genetic constitution data from 85 spelt transcripts were used to select 11 accessions, from which genes of alpha-gliadin were copied and sequenced.	High variations among accessions were observed.	[21]
To develop a tool to evaluate the immunogenic content of spelt and bread wheat gliadins.	The epitope expression levels in eleven different spelt accessions and three bread wheat accessions were measured with probes.	A wide variability in the epitope expression and accessions was observed.	[38]
To investigate gliadin epitope expression in spelt accessions and the effect of environmental factors.	121 spelt accessions were studied.	The epitope expression correlated with celiac disease varied among the spelt accessions included in the study and was not associated with environmental factors.	[22]
To detect epitope-containing peptides in T cells after ancestral (among which spelt) and common wheat ex vivo digestion using human gastrointestinal juices.	Wheat porridge of ancestral and common cereals was digested using a static ex vivo model (240 min) and analyzed with High Performance Liquid Chromatography coupled with Mass Spectrometry (ESI MS/MS).	Ex vivo digestion delivered fewer T-cell epitope-containing peptides from the ancestral wheat varieties compared to the common wheat varieties. However, ancestral wheat is still highly toxic for celiac patients.	[25]

**Table 7:** In vivo studies on the toxicity of *A. sativa* for celiac subjects with negative outcomes.

Study Details	Objective of the Study	Cereals Included	Main Outcomes	Ref.
Intervention trial, 19 adult CD patients (2 M) on a GFD.	To study the clinical setting after the consumption of oat by CD patients.	Patients received fifty g/day of oat for twelve weeks. The oats used in this trial was from a single manufacturer. The products were tested using different techniques (ELISA, western blot and mass spectrometry) resulting free of gluten contamination.	(1) Most patients tolerated oat well (apart from initial abdominal soreness and bloating); (2) one patient developed villous atrophy and dermatitis after oat consumption; (3) in 5 patients were detected positive levels of interferon γ mRNA after challenge.	[62]
Randomized, double-blind, intervention trial (from 11.3 to 14.9 months); 28 patients (age:0.7–14.2 years).	To investigate the influence of oat on the immune status of intestinal mucosa of CD patients.	Patients received either of two dietetic treatments: standard gluten free diet (GFD-std; *n* = 13) and uncontaminated gluten free diet containing oat (GFD-oat; *n* = 15). Median intake of oat was twenty g (range 3–43 g). The oat samples used in the study were specially grown, powdered, and packaged so that they were not contaminated with other toxic cereals.	A group of pediatric CD patients were not tolerant to oat. In these patients, oat affected the immune condition of intestinal mucosa: the mRNA profile suggested the presence of activated cytotoxic lymphocytes, regulatory T-cell and a stressed epithelium with altered tight junctions.	[61]
Randomized, double-blind study, 116 children with recent celiac disease diagnosis (age: 0.7–17.2 years).	To define fecal short chain fatty acids (SCFAs) profiles in children with newly diagnosed CD who received GFD with or without oat for one year.	The impact of a gluten free diet containing oat was compared to a “standard” GFD (GFD-oat, *n* = 57; GFD-std, *n* = 59). Daily oat intake (strictly gluten free): 25–50 g.	Fecal SCFAs are produced by the gut microbiota. High fecal SCFA levels in children affected by celiac disease suggest gut microflora metabolism alteration. The GFD-std group had a significantly lower total fecal short chain fatty acids levels after one year compared with 0 months ( *p* < 0.05). On the other hand, total short chain fatty acids in GFD-oat patients maintained high levels after 12 months on the gluten free diet.	[60]

GDF: Gluten free diet; CD: Celiac disease.

**Table 8:** In vivo studies on the toxicity of *A. sativa* for celiac subjects with positive outcomes.

Study Details	Objective of the Study	Cereals Included	Main Outcomes	Ref.
Randomized intervention trial. 52 adults with CD in remission; followed for 6 months (oat group: 9 M and 17 F, 48 ± 12 years; control group: 8 M and 18 F, 42 ± 10 years). Total of 40 adults with newly diagnosed CD followed for 12 months (oat group: 7 M and 12 F, 42 ± 14 years; control group: 5 M and 16 F, 48 ± 11 years).	To compare the effects of GFD with and without oat.	The consumption of oat in treated group was 50–70 g per day, taken with wheat-starch flour, muesli (60% of oat) and breakfast cereal. Oat contamination not tested.	There were not significantly differences between groups in (1) symptoms, (2) nutritional status, (3) laboratory measures. Regardless of diet, patients in remission did not show worsening architecture of the duodenal villi or increased mononuclear-cell infiltration. Except for one (control group), at one year all the newly diagnosed patients were in remission.	[64]
Self-controlled, open-labeled. Duration: 6 months. 10 children with newly diagnosed CD (5 M and 5 F); age: 6.8 ± 4.0 years.	To evaluate the safety of oat in children with newly diagnosed CD.	Patients consumed commercial oat breakfast cereal product (24 g of oat cereal/d, or 1.2 ± 0.9 g/kg/d). The gliadin content was tested using ELISA Kit. The outcomes at the end of the trial were compared with the initial evaluations (T0), without control group.	↓ biopsy score ( *p* < 0.01), ↓ intra-epithelial lymphocyte count ( *p* < 0.005), ↓ anti-tissue transglutaminase IgA antibody titer ( *p* < 0.01), ↓ number of symptoms ( *p* < 0.01).	[70]
Randomized trial. Oat group: *n* = 35 (13 M and 22 F, mean age 53 years); control group (conventional GFD): *n* = 28 (10 M and 18 F, mean age 52 (10) years).	To evaluate the safety of long-term inclusion of oat in celiac patients’ diets.	Both groups followed a GFD for 5 years. Treated group were allowed to eat oat freely. The oat products were gluten-free.	No significant differences between groups in (1) duodenal villous architecture, (2) inflammatory cell infiltration of the duodenal mucosa, (3) antibody profile.	[63]
2-year intervention study. 20 adult patients (age: 22–71 years) (5 drop-out during the study).	To investigate the safety of the long-term inclusion of oat in the diet of CD adult patients.	Median daily intake of oat: 93 g. The gluten contamination of oat products (rolled oat) was tested using ELISA Kit.	No adverse effects were observed in (1) small bowel histology, (2) serology, (3) nutritional status.	[68]
1-year randomized intervention trial, 116 CD children.	To evaluate the possible negative effects of oat in some CD patients.	Patients were randomized to 1) GFD-std: a standard GFD, 2) GFD-oat: a GFD supplemented with oat. The urinary nitrite/nitrate concentrations were monitored at 0, 3, 6, 9 and 12 months.	No significant differences were observed.	[73]
1-year double blind multicenter study. 116 children (mean age: 6.5 years, range 8 months–17.5 years; M:F distribution 1:1.4); 92 participants complete the study.	To evaluate if children with CD tolerated oat in their GFD.	Subjects were randomized in two groups (1) GFD-std (n = 50): follow a standard GFD, (2) GFD-oat (n = 42): follow a GFD with additional oat products (wheat free). Median of oat intake was 15 g/day. The oats used were specially grown, milled, and packaged and the gluten contamination was tested using ELISA Kit.	No significant differences between groups in (1) serological markers, (2) small bowel mucosal architecture, (3) numbers of intraepithelial lymphocytes.	[72]
2-year controlled trial. 32 children with celiac disease: *n* = 13 oat challenge: 6 F, 7 M, median age 11 (9–17) years; *n* = 10 gluten challenge: 9 F, 1 M, median age 13 (7–15) years; *n* = 9 newly detected CD, GFD with oat, median age 12 (8–14) years.	To evaluate the long-term safety of oat in children with CD.	23 children in remission were randomized either to oat (50 g/day) or gluten (20 g/day) group; when small bowel histological relapse was evident after gluten challenge, a GFD including oat was started. 9 newly detected celiac patients followed an oat-containing GFD. The rolled oat provided during the trial were tested with ELISA assay and polymerase chain reaction techniques and were free from contamination.	In celiac children in remission, oat had no detrimental effect on the parameters considered during the 2-year trial (intestinal histology or serology). On the contrary, the gluten-challenge group relapsed after 3–12 months. All patients (relapsed or newly detected) with an oat-containing GFD, showed a complete recovery from the disease.	[71]
5-year follow-up intervention study. 42 CD patients, oat group: 22 patients (10 drop-out during the study), control group: 20 patients.	To clarify the long-term inclusion of oat in the GFD by analyzing local cellular immunological responses.	Median daily intake of oat: 30 g (range 10–70 g). The purity of the oat-products was confirmed during the trial.	Long-term consumption of oat does not stimulate an immunological response locally in the mucosa of the small intestine of CD patients.	[69]
2-year follow-up intervention study. 23 CD patients (7 F; 7–18 years).	To study the toxicity of oat in CD children.	Patients were randomized to (1) oat challenge, (2) gluten challenge (grains with gluten and oat). Patients in gluten group continued only with oat after jejunal histological relapse was evident. The uncontaminated oat products (cultivar not indicated) were given to the patients (processed as rolled oats).	No significant change was observed in the intensity of TG2-targeted autoantibody deposits in the oat group, within 2 years. The intensity of deposits in the gluten containing grains group clearly decrease after the exclusion of gluten, despite oat consumption.	[74]
Prospective study. 15 adults with CD (age: 57 ± 9 years)	To test the safety of oat products manufactured under the Canadian Celiac Association guidelines.	Subjects were tested with 350 g/week of pure oat for 12 weeks. Pure, uncontaminated oats, tested with ELISA assay, were provided to patients.	During oat consumption no significant changes were observed in symptom scores, weight, hemoglobin, ferritin, or albumin and histology scores. tTG remained negative in all patients.	[65]
Cross-sectional follow-up study. 106 long-term treated CD adults. Oat group: *n* = 70 (median age: 59 (24–81) years), no oat group: n= 36 (median age: 54 (36–73) years).	To evaluate the long-term tolerability of oat for CD patients.	Median oat consumption: 20 g/d (1–100 g/d) for up to 8 years (oat market products).	Oat consumption was not determined: (1) small-bowel mucosal villous damage, (2) inflammation, (3) gastro-intestinal symptoms.	[66]
Intervention study. 58 F and 15 M with CD (aged 20–69 years, median 51).	To evaluate whether ingestion of oat stimulate an avenin specific T cell response in vivo.	Patients consumed for 3 days 100 g per day dry weight oat prepared as porridge. Three sources of oats were used. Two of them were labelled as having gluten content <3 ppm.	Avenin-specific responses were observed in 8% patients. In vitro, immunogenic avenin peptides were susceptible to digestive endopeptidases and showed a reduce HLA-DQ2.5 binding stability. The low rates of T-cell activation after an oat challenge (100 g/d) supports the safety of long-term oat consumption at the doses commonly consumed.	[67]
Randomized double-blind, placebo-controlled, crossover multicenter study. 15-month trial, 177 children (4–14 years of age) with CD, on a GFD for ≥2 years.	To evaluate the long-term safety of oat in the treatment of children with CD.	Children consumed gluten-free food containing an age-dependent amount (15–40 g) of either placebo or purified nonreactive varieties of oat (Irina and Potenza) for 2 consecutive 6-month periods separated by washout standard GFD for 3 months.	Direct treatment effect was not statistically significant for clinical, serologic, and intestinal permeability variables.	[75]

GFD: Gluten free diet; CD: Celiac disease; tTG: Tissue transglutaminase.
